# Fabrication and Conductivity of Graphite Nanosheet/Nylon 610 Nanocomposites Using Graphite Nanosheets Treated with Supercritical Water at Different Temperatures

**DOI:** 10.3390/polym14214660

**Published:** 2022-11-01

**Authors:** Jun-Ven Lim, Soo-Tueen Bee, Lee Tin Sin, Chantara Thevy Ratnam, Zuratul Ain Abdul Hamid

**Affiliations:** 1 Department of Mechanical and Material Engineering, Lee Kong Chian Faculty of Engineering and Science, Universiti Tunku Abdul Rahman, Jalan Sungai Long, Bandar Sungai Long, Cheras, Kajang 43000, Malaysia; 2 Department of Chemical Engineering, Lee Kong Chian Faculty of Engineering and Science, Universiti Tunku Abdul Rahman, Jalan Sungai Long, Bandar Sungai Long, Cheras, Kajang 43000, Malaysia; 3 Radiation Processing Technology Division, Malaysian Nuclear Agency, Bangi, Kajang 43000, Malaysia; 4 School of Materials and Mineral Resources Engineering, Engineering Campus, Universiti Sains Malaysia, Nibong Tebal 14300, Malaysia

**Keywords:** conductivity, graphite nanosheets, polymer nanocomposites, supercritical water treatment

## Abstract

In this study, water at high temperatures (150, 175, 200 °C) and in a vacuum state (−0.1 MPa) was applied to graphite nanosheets to enhance surface activity to promote the formation of oxygen-containing functional groups through supercritical water treatment. Nylon 610 nanocomposites (with treated or untreated nanosheets as nanofillers) were then synthesized using interfacial polymerization. X-ray diffraction (XRD) analysis showed that the water treatment did not alter the crystal structure of the carbon nanosheets. Additionally, Fourier transform infrared spectroscopy (FTIR) analysis showed the presence of amide peaks within the nanocomposites, indicating the presence of hydrogen bonding between the nanosheets and the polymer matrix. The intensity of the amide peaks was higher for nanocomposites combined with treated nanosheets than untreated ones. This hydrogen bonding is beneficial to the conductivity of the nanocomposites. The conductivity of treated nanosheets/nylon nanocomposites generally decreased with increasing wt%, while the conductivity of untreated nanosheets/nylon nanocomposites increased with increasing wt%. The decrementing of conductivity in the treated nanosheets/nylon nanocomposites is due to the agglomeration of the nanosheets within the composite. This is in in line with scanning electron microscopy (SEM) results which showed that at higher wt%, the aggregation condition tended to occur. The highest conductivity obtained is 0.004135 S/m, as compared to the conductivity of neat nylon 610, which is 10^−14^ S/m. This improvement in electrical properties can be attributed to the intact structure of the nanosheets and the interaction between the nanofillers and the nylon 610 matrix. The optimum nylon 610 nanocomposite synthesized was the one incorporated with 0.5 wt% graphite nanosheets treated at 200 °C and −0.1 MPa, which possess the highest conductivity.

## 1. Introduction

Nylon is known for its wide engineering applications; however, its electrical insulating properties remain an obstacle preventing it from being used more widely, especially for applications in environments where conductivity is required [1,2]. Nylon’s ubiquitous application is due to some of its excellent properties such as high mechanical strength, excellent thermal resistance, good fatigue and wear resistance and moderate cost. One of the methods used to overcome the insulating properties of nylon is to incorporate carbon nanofillers such as carbon nanotubes [3], graphene oxides [4] and fullerenes [5] into the nylon matrix to produce a nylon/graphite composite. Thus, in the last decade, graphene-based nanostructures have been extensively studied as part of a novel generation of composite materials [3,4,5,6]. The reason these nanoscale materials were chosen as nanofillers was due to their outstanding mechanical properties, extraordinary surface area and conducting behaviour [7]. Graphite nanosheets, which are composed of layers of graphene sheets attracted to each other by van der Waals forces [8], were chosen because of their affordability and wide availability. Graphene nanosheet remains one of the few modified graphene substances to be manufactured at the level necessary for composite materials and structural applications [9]. The addition of a small amount of nanofiller can also lead to a significant improvement in mechanical properties. A large amount of research has been performed in order to discover how carbon nanofillers affect the electrical behaviour of semi-crystalline polymers. The most effective method used to enhance the properties of an original polymer is the introduction of better surface interaction. One of the approaches to achieve this involves introducing functional groups on the surface of the nanofillers in order to interact with the interphase of the polymer matrix. In this research, supercritical water treatment was performed in order to modify the surface of the graphite nanosheets, specifically by activating oxygen-containing active groups on its surface, such as hydroxyl, carbonyl or carboxyl groups.

Supercritical water exists when water has reached a temperature and pressure above the thermodynamic critical point (at 373 °C and 22.1 MPa) [10]. Beyond this critical point, the liquid and vapour phases can coexist and are indistinguishable from one another. Under these conditions, supercritical water obtains some unique properties. For example, the fluid can act differently as a solvent compared to regular water, as supercritical water possesses low viscosity along with excellent diffusion and solubility abilities [11]. Additionally, supercritical water possesses superb oxidation ability due to the oxygen and water forming a homogenous phase together and becoming a superior fluid with high solubility for oxygen and organic substances [12]. This makes supercritical water suitable for activating oxygen-containing functional groups on the surface of graphite nanosheets. The increase in surface activity of the carbon nanofillers also allows them to be easily dispersed in water. This stable dispersion helps facilitate the fabrication of nanocomposites with nanosheets as nanofillers, as reported in the research of Wang et al. [13]. Therefore, supercritical water has the potential to be used in the preparation of many other polymer composites. The reason for focusing on supercritical water in this research over other supercritical solvents like carbon dioxide (CO_2_) or toluene is that water is easily available, inexpensive, recyclable and relatively safe to handle. Supercritical water treatment is generally performed by adding distilled water and the nanofillers into a reaction vessel. The vessel or reactor is then heated to the supercritical temperature of 373 °C, and the pressure is raised to 22.1 MPa.

Furthermore, in this research, the temperature of the supercritical water is manipulated in order to determine the effect of different temperatures on the treatment of the nanosheets. This is because, while there are a few studies that have focused on the result of supercritical fluid treatment of nanofillers, few have reported on the effect of using a supercritical fluid at different temperatures and pressures. This can also be useful to determine whether a lower temperature can achieve the same effect that water in supercritical condition has, thus helping to save energy. The microstructures of graphite nanosheets/nylon composites were observed, and the conductivity of the nanocomposites was investigated in detail. The purpose of this work is to report a convenient method for the preparation of the graphite nanosheets/nylon nanocomposites, which can be applied to aid materials engineering design for the development of new nanomaterials.

## 2. Materials and Methodology

### 2.1. Materials

In this research, graphite nanosheets (with a particle diameter of 2–10 nm) were used as nanofillers and were manufactured by Qingdao Tiansheng Graphite Co., Ltd. (Qingdao, China) in China. The nanosheets were also the only reinforcing filler for this research. The purity of the nanosheets is labelled as ≥99%. The hexamethylenediamine (HMDA) and sebacoyl chloride that were used as reactants to synthesize the nylon 610 polymer were supplied by Merck Sdn Bhd and had a purity of ≥99%. The hexane that was used as a base to dissolve the sebacoyl chloride, along with the sodium hydroxide (NaOH) used to neutralise the hydrogen chloride that arose from the polymerization reaction, were also supplied by Merck Sdn Bhd in Subang Jaya, Malaysia. The purities of the hexane and NaOH were ≥99%.

### 2.2. Preparation of Nylon 610/Graphite Nanosheets Composites

Firstly, to prepare supercritical water-treated graphite nanosheets, the desired amount of nanosheets and 100 mL of distilled water were added to a 250 mL beaker. The mixture was then stirred using a homogenizer for 10 minutes to evenly distribute the nanosheets in the water.

**Figure 1:**
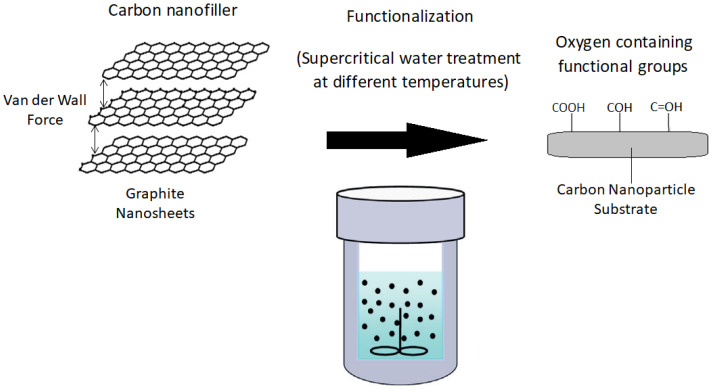
Supercritical Water Treatment Process of Graphite Nanosheets.

After that, the carbon nanosheets and distilled water were placed in a vacuum oven, where the temperature and pressure could be controlled. The pressure in the vacuum oven was maintained at a vacuum state (−0.1 MPa) while the temperature of the oven was kept at 200 °C for a period of 30 min. At this state of pressure and temperature, the distilled water was transformed into supercritical water. After the supercritical water treatment was complete, the pressure and temperature were reduced to atmospheric pressure and room temperature, respectively. Such steps of treating carbon nanosheets with supercritical water were repeated 5 times at different graphite loadings (0.5, 1.5, 2.5, 3.5 and 4.5 wt%) for mixing with nylon 610. The reason for the selection of those graphite loading levels was because nanofillers merely require a small amount (less than 5 wt%) to be incorporated into the polymer to deliver noticeable enhancements to the properties of the nanocomposites. Such levels of loading for graphene nanosheets have been reported by Wang, Li and Wu [14]. Most nanofillers have a large surface area-to-volume ratio. Meanwhile, the supercritical water treatment was also repeated using different temperatures of 175 °C and 150 °C with the same 5 levels of graphite loadings. The supercritical water treatment process is illustrated in Figure 1.

**Figure 2:**
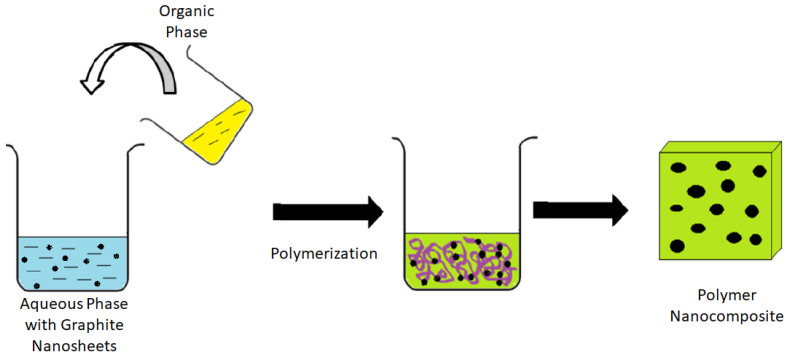
Interfacial Polymerization of Nylon 610/Graphite Nanosheets Nanocomposite.

The synthesis of nylon 610/treated carbon nanosheet composites by interfacial polymerization was carried out by preparing an aqueous phase and an organic phase. The aqueous phase comprised 6.0 g (7.143 mL, 51.63 mmol) of HMDA and 2.0 g (0.5 M, 50 mmol) of NaOH added to 100 mL of distilled water with the supercritical water-treated carbon nanosheets in it. The organic phase consisted of 2.0 mL (2.24 g, 9.367 mmol) sebacoyl chloride in 100 mL hexane. After these two solutions were prepared, the organic phase was then carefully poured on top of the aqueous phase using a glass rod (because the organic phase was less dense than the aqueous phase). Then, a film of nylon 610/treated nanosheet formed at the interface. The film was grasped with tweezers and raised as a rope of continuously forming nylon 610/treated carbon nanosheet film onto a metal plate. The nylon 610/treated carbon nanosheet composite was then washed well with distilled water and then soaked in distilled water for 30 min to remove any remaining chemicals. This was followed by drying in an oven at 60 °C for 24 h. This step was repeated for the different graphite loadings. Additionally, to determine the difference between polymer nanocomposites that used treated carbon nanosheets and those that used untreated nanosheets as nanofillers, the above steps were repeated; however, the carbon nanosheets were not treated with supercritical water but added to nylon 610 in its original state. The synthesis of nylon 610/graphite nanosheet nanocomposites is illustrated in Figure 2.

### 2.3. Characterization Testing

#### 2.3.1. Conductivity Test

The electrical conductivity properties of the nylon 610/graphite nanosheets nanocomposites were measured via a potential state meter using the two-point probe method. The reason the two-point probe method was used in this work was because this work was dealing with a highly non-conductive polymer matrix; the four-point probe method is generally used for highly conductive materials, where the method helps in ensuring a more accurate reading of resistance by eliminating contact resistance between the metal probe and the sample [15,16]. This potential state meter was operated by applying a current to two probes (with a distance of 2 cm between them) and then measuring the resultant voltage drop. A current was applied to each of the nanocomposite samples from 0.01 to 0.1 A with 50 steps in between and a settling time of 1s for each of the steps, as done in the research of Postiglione et al. [17]. The conductivity of the samples was then calculated using resistance measurements. The resistance of the nanocomposite can then be calculated using Equation (1) below [18]:

(1) ρ=[πt/ln2]×(V/I)

where *t* is the thickness of the sample, *V* is the voltage, *I* is the current measured, and *ρ* is resistance, with conductivity (*σ*) being *σ* = 1/*ρ*.

Specifically, the equation above calculates the sheet resistance of a sample, which is typically used for thin films of conducting and semiconducting materials. The condition for using this equation is that the thickness of the sample has to be less than the spacing between the probes (2 cm) [19]. The thicknesses of the samples were measured using Vernier callipers, and the thicknesses of all of the nanocomposites were found to be around 2 mm. One important advantage that sheet resistance possesses when compared to other resistance measurements is that it is not dependent on the size of the sample (only the thickness), which allows differently shaped samples to be easily compared with one another [20]. Additionally, the resistance of each nanocomposites sample was taken as an average of 5 specimens. The resistance and conductivity data for each of the 5 samples for all nanocomposites synthesized can be found in Appendix A in Table A1, Table A2, Table A3, Table A4, Table A5, Table A6, Table A7, Table A8, Table A9, Table A10, Table A11, Table A12, Table A13, Table A14, Table A15, Table A16, Table A17, Table A18, Table A19 and Table A20.

#### 2.3.2. X-ray Diffraction (XRD) Test

X-ray diffraction analysis (XRD) testing was performed to obtain information about the dispersion state of the graphite nanosheets in the nylon 610 polymer matrix using a Shimadzu XRD 6000 X-ray diffractometer. The XRD spectra of the nylon 610/graphite nanosheets composites were recorded with the diffractometer using a Cu-Kα radiation generator that had a wavelength of 1.542 Å, while the rotational sample stage had a measuring angle range of 2*θ* = 5° to 40° and a constant scanning rate of 1.2° min^−1^. The operating current and acceleration voltage of the Cu-Kα radiation generator were set at 30 mA and 40 kV, respectively. The interlayer spacing, or *d*-spacing, *d* of crystallites was calculated using Bragg’s equation, which is shown in Equation (2) [21]. Additionally, the inter-chain separation *R* of crystallites was determined by using the Klug and Alexander equation, as shown in Equation (3) [21]:

(2) $$d=\frac{\lambda }{2\;\sin\theta }$$

(3) $$R=\frac{5\lambda }{8\sin\theta }$$

where *λ* is 1.542 Å, and *θ* is the Bragg angle in radians.

#### 2.3.3. Fourier Transform Infrared Spectroscopy (FTIR) Analysis

Fourier transform infrared (FTIR) spectroscopy was performed in order to analyse the different functional groups and chemical bonds within the graphite nanosheets/nylon 610 nanocomposites by using a Nicolet IS10 FTIR spectrometer. The nanocomposite samples were first placed at the centre of the attenuated total reflectance (ATR) plate. Then, the ATR press was lowered and pressed on top of the sample. After that, the dial attached to the lowered ATR press was turned around until a “click” sound could be heard. The samples of the composites were scanned under a band region of 4000 cm^−1^ to 400 cm^−1^.

#### 2.3.4. Scanning Electron Microscopy Analysis (SEM)

SEM analysis was performed to analyse the surface morphology of the graphite nanosheets/nylon 610 nanocomposites using a Hitachi S-3400N scanning electron microscope. Firstly, the nanocomposite samples were broken in half, and the fractured surface of the nanocomposite was then placed facing up on top of a carbon tape. The tape was located on an aluminium stub that had a diameter of 10 mm. The aluminium stub was then coated in gold and palladium (via the EMITECH SC7620 sputter coater) to obtain the highest-quality images and to prevent charging of the samples. The coated samples were then sent to the SEM chamber and scanned with an electron beam with a voltage of 15 kV. The SEM micrographs were then recorded at a magnification of 3000 times.

## 3. Results and Discussion

### 3.1. Electrical Conductivity

**Figure 3:**
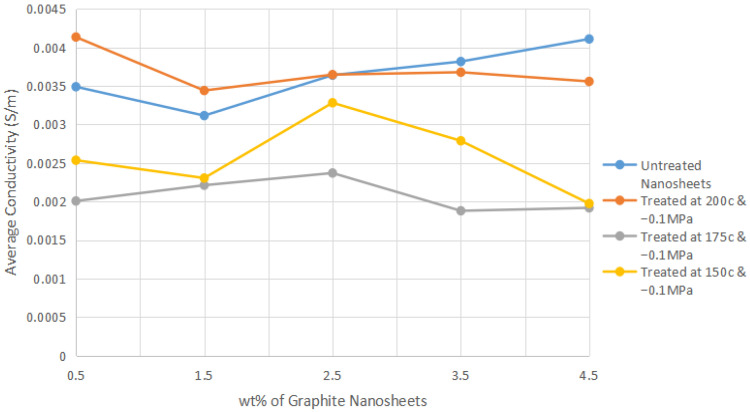
Average Conductivity of All the Nylon 610/Graphite Nanosheets Nanocomposites.

Firstly, percolation theory states that electricity is conducted through a polymer matrix when electrons are free to move between the electrically conductive nanofillers [22]. Thus, the conductivity of the polymer nanocomposite can be determined by the amount of contact between the conductive nanofillers, as this contact can form a continuous path that allows the electrons to flow through the polymer composite matrix [23]. This path is known as a conductive network, and any substance containing these networks is capable of conducting electricity; according to percolation theory, the point where a continuous conducting cluster is formed is where the percolation threshold is reached, and the resistivity of the material will decrease greatly at that point [22]. Additionally, it is also important to comprehend the factors that can affect the percolation threshold so as to better utilize a material as conductive filler, such as the filler concentration and the characteristics of the filler, including size, shape and surface morphology. The above factors can be used to explain the patterns of conductivity for the nanocomposites seen in Figure 3.

**Figure A1:**
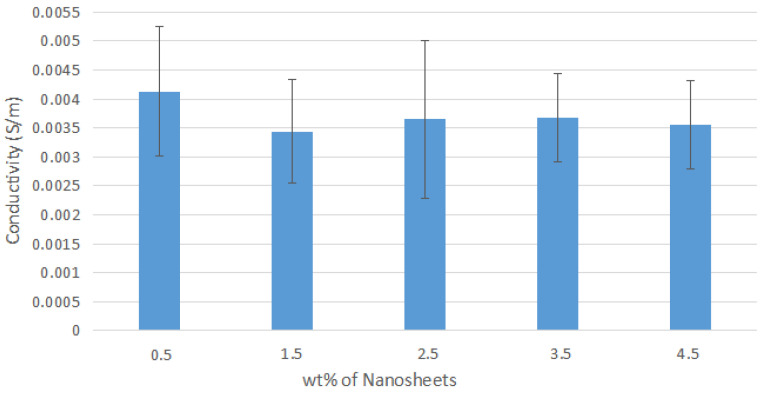
Average Conductivity of Nylon 610 Nanocomposites with Nanosheets Treated at 200 °C and −0.1 MPa.

**Figure A2:**
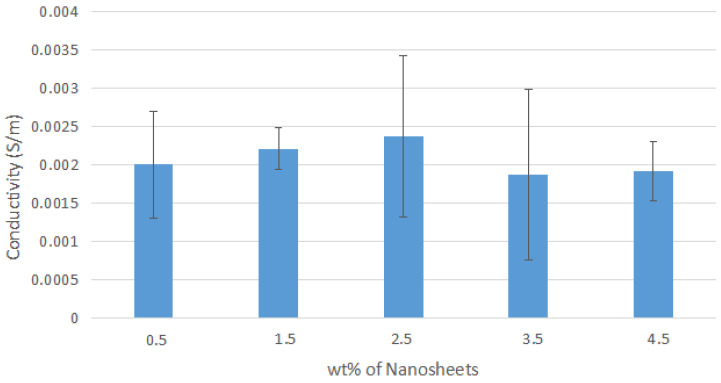
Average Conductivity of Nylon 610 Nanocomposites with Nanosheets Treated at 175 °C and −0.1 MPa.

**Figure A3:**
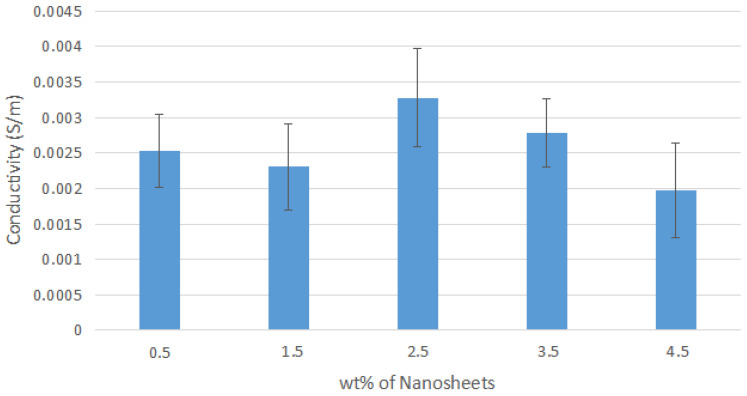
Average Conductivity of Nylon 610 Nanocomposites with Nanosheets Treated at 150 °C and −0.1 MPa.

**Figure A4:**
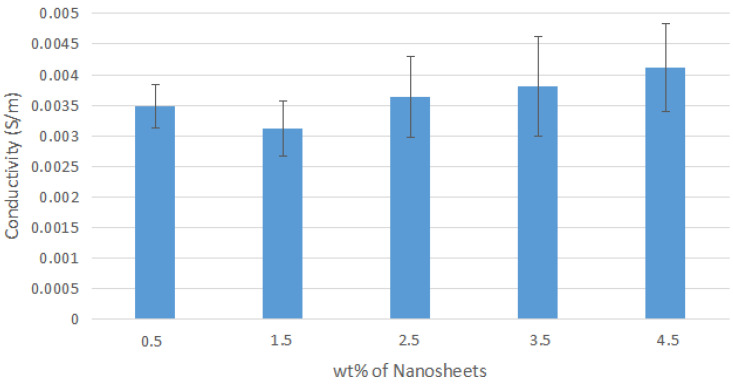
Average Conductivity of Nylon 610 Nanocomposites with Untreated Nanosheets.

The graph of the average conductivity of nylon 610/graphite nanosheets composites with respect to the different amounts of graphite nanosheets used as nanofillers is displayed in Figure 3; the bar chart with error bars for each of the 20 samples can be found in Appendix A in Figure A1, Figure A2, Figure A3 and Figure A4. The individual conductivities for all 20 of the nanocomposites are tabulated in Table 1. It can be seen from the above figure that the increase in loading level of the untreated nanosheets resulted in a general improvement in conductivity for the nanocomposites. This can be seen in the incorporation of 4.5 wt% untreated nanosheets, which resulted in the second-highest conductivity (0.004110 S/m) out of all of the nanocomposites synthesized. This is due to the fact that an increase in concentration of the filler will lead to a higher chance of forming a conductive network, as filler particles need to be in contact with one another to form the network, as explained in the percolation theory. Furthermore, additional increasing of the filler resulted in the increase of the cross section of the network due to the formation of more parallel pathways, which further reduced the resistivity of the nanocomposites [24]. This explains why the addition of 4.5 wt% untreated nanosheets resulted in the highest conductivity out of all of the untreated nanosheets/nylon 610 nanocomposites.

However, the opposite can be seen occurring for the incorporation of treated nanosheets, as the addition of these nanofillers decreased the average conductivity of the nanocomposites with an increase in the loading levels of the treated nanosheets, noticeably starting from 2.5 wt% onwards. The reason for this reduction in conductivity might be the change in the surface morphology of the treated nanofillers, which is the formation of oxygen-containing functional groups on the surface of the nanosheets after supercritical water treatment, as this is not conducive to the flow of electricity [13]. This has also been shown by Park et al. [25], where the conductivity of a functionalized graphene nanofiller is related to the number of defects on its surface that are formed after an oxidation–reduction process. Although it is worth noting that even the lowest conductivity out of all the nanocomposites (0.001880 S/m) is a significant increase when compared to neat nylon 610, which has an average conductivity of 10^−14^ S/m, as seen in industry-made nylon 610 that is unreinforced and in a resin state and articles that researched nylon 610 nanocomposites [26]. Another reason for this trend may be the aggregation of the nanofillers, even though functionalization of carbon nanofillers is known to reduce agglomeration and enhance their dispersion in solvents [27]. However, the high concentration of fillers may negate any benefits that functionalization provides and subsequently present a higher probability of the fillers aggregating due to the high amount of fillers in the polymer matrix. This explains why an increasing amount of nanofillers negatively affected the conductivity when treated nanosheets were incorporated into the nanocomposites, whereas the opposite condition occurred for untreated nanosheets due to the formation of oxygen-containing functional groups on the nanosheets after water treatment. These functional groups have a greater chance to form bonds with other groups on different treated nanosheets when they are present in large amounts. As a result, the treated nanosheets form aggregates, which causes lower conductivity. The reason why aggregation negatively affects conductivity is that the aggregates have lower surface area (as they are clumped together), so lower amounts of conductive fillers are in contact with one another, and fewer paths exist for electron transport, thus decreasing the conductivity.

Additionally, there is also a discrepancy found within the conductivity patterns mentioned above, which is: for most of the nanocomposites, the conductivity decreased from 0.5 wt% to 1.5 wt% before increasing at 2.5 wt%, even though an increase in conductive nanofiller content is correlated to increasing conductivity. This observation can be explained by the fact that at a low loading level, there are insufficient nanofillers to form an effective conductive network, as the nanofillers are not distributed very evenly in the polymer matrix, and as a result they are unable interact among themselves. Additionally, it could be due to the forces and groups within the nanosheets that cause reactions, leading to aggregation even in relatively low amounts, such as the oxygen-containing groups formed after water treatment in treated nanosheets and the van der Waals forces found in untreated nanosheets.

According to Figure 1, it is also observed that the temperature of the water used to functionalize the carbon nanosheets during the supercritical water treatment has an effect on the conductivity of the nanocomposites that incorporate them, as the average conductivity decreases as the temperature of the water used drops from 200 °C to 150 °C to 175 °C. The explanation for this phenomenon is that supercritical water has the ability to moderately oxidize the edges and surfaces of the carbon nanosheets [12], and using a lower temperature during this water treatment for the same amount of time has the effect of retaining more of their graphite-like properties, as the nanosheets are not properly oxidized [28]. This unfavourably affected the conductivity, as it is the slight oxidation of the nanosheets and the formation of polar functional groups (carboxylic, carbonyl or hydroxyl groups) on their surfaces that helps enhance contact with the polymer matrix due to the newly formed functional groups, thus increasing the electrical conductivity of the nanocomposites [13]. This is evident in the highest conductivity among all the nanocomposites synthesized belonging to the one incorporated with 0.5 wt% treated nanosheets treated at 200 °C (0.004135 S/m). This indicates that the higher the temperature of the supercritical water treatment, the higher the oxidation level of the nanosheets. However, Figure 3 also shows that the nanocomposites combined with nanofillers treated with water at 175 °C have a lower conductivity than those combined with nanofillers treated with water at 150 °C. The reason for this discrepancy is that at the lower temperature of 150 °C, the water treatment is not enough to promote oxygen functionalities on the surface of the nanosheets, leading to the nanosheets retaining more graphite-like characteristics such as high conductivity. Also, the supercritical water treatment at 175 °C of the nanosheets led to an unfortunate middle ground, in that the treatment did not give rise to enough functional groups on the sheets’ surface to interact with the polymer matrix, while the sheets retained many of their original graphite qualities due to oxidation, thus resulting in these nanocomposites having the lowest conductivity overall.

### 3.2. XRD Analysis

**Figure 4:**
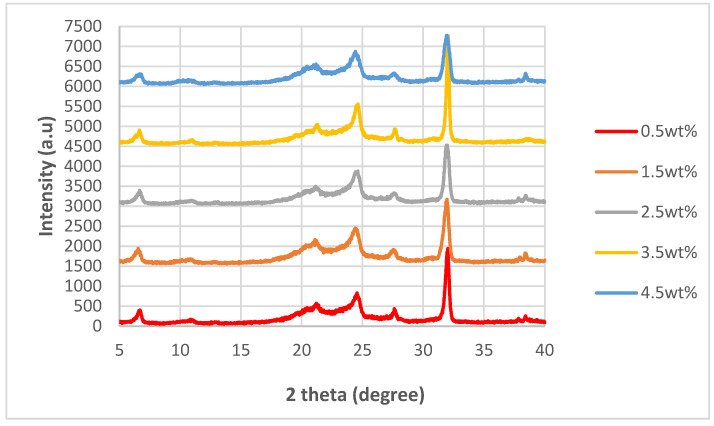
XRD Spectra of Nylon 610 Nanocomposites with Graphite Nanosheets Treated at 200 °C and −0.1 MPa.

**Figure 5:**
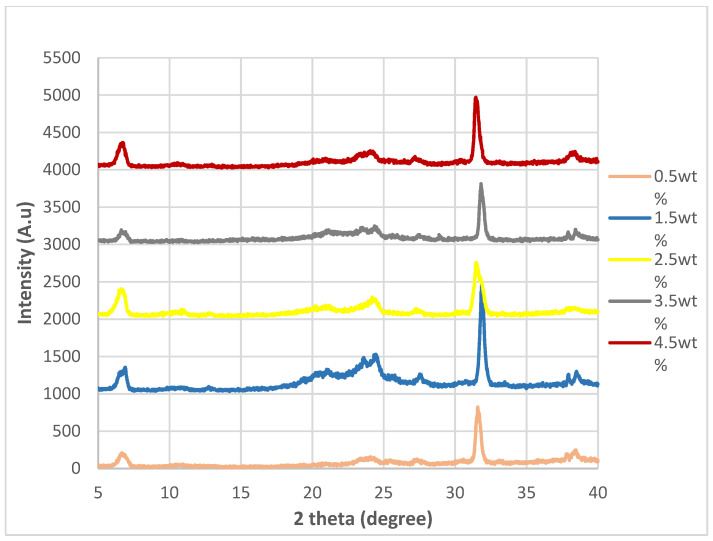
XRD Spectra of Nylon 610 Nanocomposites with Graphite Nanosheets Treated at 175 °C and −0.1 MPa.

**Figure 6:**
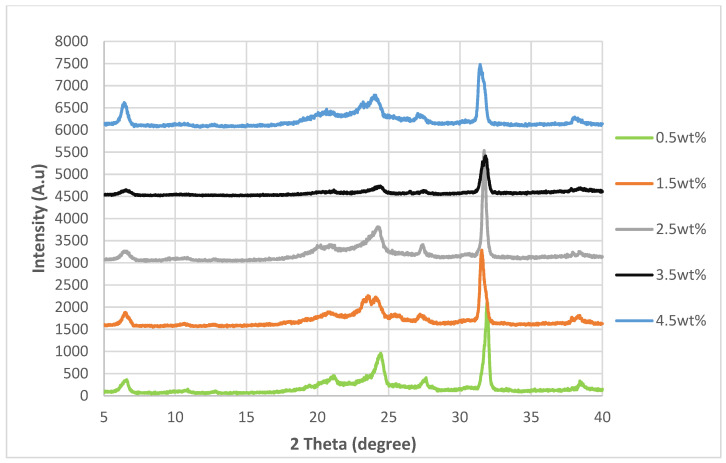
XRD Spectra of Nylon 610 Nanocomposites with Graphite Nanosheets Treated at 150 °C and −0.1 MPa.

**Figure 7:**
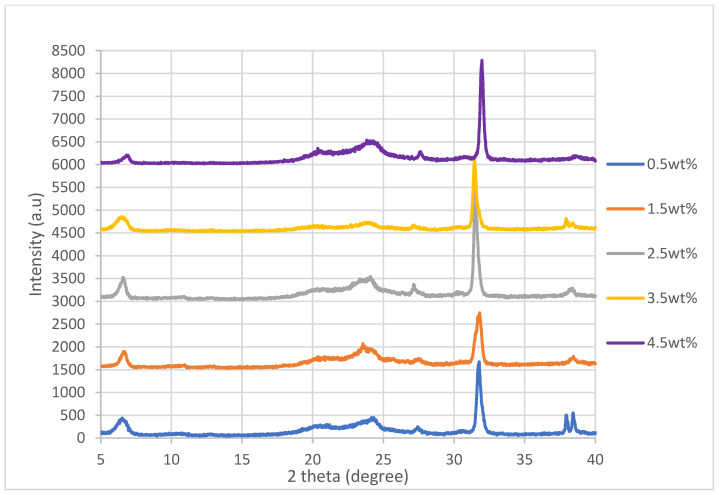
XRD Spectra of Nylon 610 Nanocomposites with Untreated Graphite Nanosheets.

**Figure 8:**
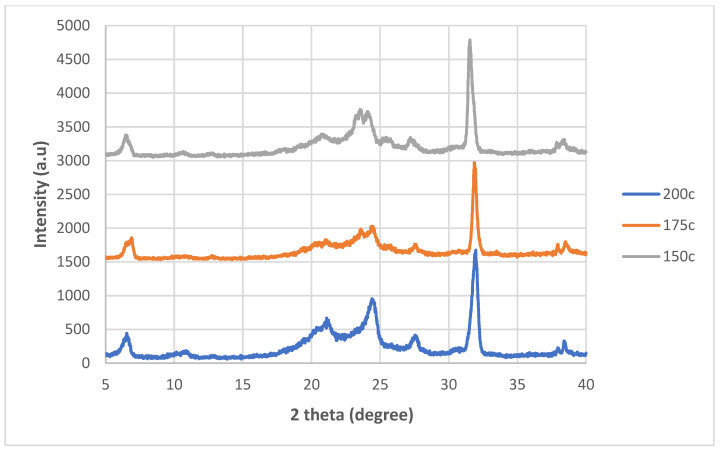
XRD Spectra of Spectra for Nylon 610 Nanocomposites with 1.5 wt% Graphite Nanosheets Treated at All Temperatures for Supercritical Water Treatment.

The XRD spectra for the nanocomposites incorporated with untreated nanosheets and nanosheets treated at 200 °C, 175 °C and 150 °C while at −0.1 MPa are shown in Figure 4, Figure 5, Figure 6 and Figure 7. By referring Figure 4, Figure 5, Figure 6 and Figure 7, it can be shown that there are two relatively strong peaks at approximately 2*θ* = 21.1° and 24.3°, and two short shoulders at around 2*θ* = 20.5° and 27.0° for all 20 of the nanocomposites. The peaks at 2*θ* = 20.5°, 21.1° and 24.3° are characteristic peaks of nylon 610, with 2*θ* = 20.5° and 24.3° corresponding to the α-crystalline form of the nylon with crystallographic reflections of (100) and (010/110) [29,30]; while the peaks at 21.1° refer to the γ-crystalline form with reflections of (001) [31]. Furthermore, the weak peak at 2*θ* = 27.0° is associated with the graphite diffraction plane of (002) [32], confirming the presence of graphite nanosheets in the polymer matrix and the element of carbon [33]. While the overall structure of the XRD spectra remains similar for all of the nanocomposites analysed, there are still some noticeable differences between the nanocomposites that incorporated either untreated or treated nanosheets, one of them being that the peaks for 2*θ* = 20.5°, 21.0°, 24.3° and 27.0° in all the untreated nanosheets/nylon 610 composites and composites with nanosheets treated at 175 °C and 150 °C are broader and have much lower intensity. This suggests that the nanocomposites made with untreated nanosheets and nanosheets treated at a lower temperature have a smaller size than the ones combined with nanosheets treated at 200 °C, as Scherrer’s equation states that the broader the width of the peaks, the smaller the particle size. The reason behind this is that the intercalation effect of the supercritical water treatment and the formation of oxygen functional groups may increase the size of the nanosheets in the polymer matrix, which is enhanced at higher temperatures. This is also supported by Figure 8, which shows the XRD spectra of nylon 610 nanocomposites with 1.5 wt% treated nanosheets at all temperatures for supercritical water treatment. This is shown in the peaks for the nanocomposite with nanosheets treated at 200 °C, as they are narrower than those with nanosheets treated at 175 °C and 150 °C.

While most of the peaks in the XRD graph are comparable to those of other nylon/carbon nanofiller nanocomposites, there are still some disparities between them. For example there is a peak at approximately 2*θ* = 7.0°. While there is also a similar peak that occurs in the wide-angle X-ray diffraction (WAXD) pattern of graphite oxide, the diffraction peak is at around 10°. The presence of this strong peak was explained to be a result of oxygen-containing functional groups forming chemical bonds between graphite layers during oxidation [34]. The possible reason behind the decrease to a lower angle might be that the oxidation of the graphite nanosheets by the water treatment was not thorough enough, as the temperature of the water was below supercritical condition, with the surface of the water-treated nanosheets being covered with fewer oxygen functionalities when compared to that of graphite oxide.

**Figure 9:**
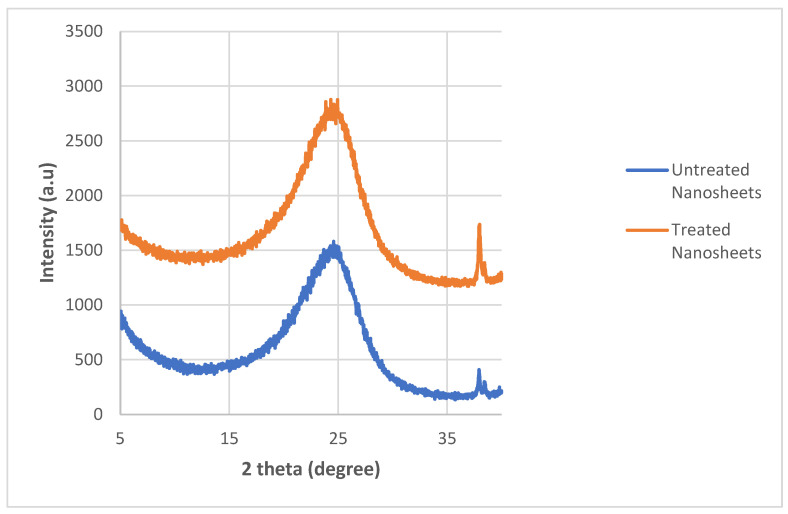
XRD Spectra of Treated and Untreated Graphite Nanosheets.

The XRD spectra for both treated and untreated graphite nanosheets are shown in Figure 9. Both of these spectra displayed graphite diffraction peaks of the (002) plane at 24.6°, which is similar to that of graphene oxide as shown in the works of Gupta et al. [35]. The presence of a broad peak in both treated and untreated nanosheets indicates that both of these structures are composed of layers of nanosheets, signifying the presence of multilayer domains [36]. Moreover, after the water treatment, it was found that the *d*-spacing had increased from 3.6105 Å to 3.6597 Å (as seen in Table 2), which was mainly due to the intercalation with water and formation of oxygen-containing functional groups on the surface of the nanosheets, which resulted in an increase of the inter-chain separation. On the other hand, the XRD patterns for the treated and untreated nanosheets indicate that the shapes of the diffraction peaks do not differ much from one another. A similar phenomenon has also been observed by Wang et al. [13]. Thus, it can be concluded that the supercritical water treatment has no effect on the crystal structure of the graphite nanosheets. The enhancement of conductivity in the graphite nanosheets/nylon 610 composites can also be attributed to the intact crystal structure of the graphite nanosheets, due to their inherent conductivity. There is also a discrepancy between the XRD spectra of the nanosheets and pure graphite, in that the intense peak for the graphite diffraction plane has shifted from 27.0° (as seen in pure graphite) to a broad peak at 24.6°, which might be due to the presence of oxygen functionalities on the nanosheets. The small peaks near 38.0° for both untreated and treated nanosheets might be due to impurities within the nanosheets.

Furthermore, the 2*θ*, *d*-spacing and inter-chain separations for all the 2*θ* = 24.3° peaks of the graphite nanosheets/nylon 610 nanocomposites were tabulated in Table 3. Generally speaking, from the table below, it can be seen that increasing the amount of nanosheet particles from 0.5 wt% to 1.5 wt% resulted in an increase in the *d*-spacing and inter-chain separation for the deflection peaks of the nanocomposites, which can be attributed to the good dispersion of nanosheets within the polymer matrix. However, the *d*-spacing and inter-chain separation was slightly reduced when the loading level of nanosheets was increased to 2.5 and 3.5 wt% loading levels before demonstrating a drop at 4.5 wt%. The decrease in spacing can be explained by the poor interaction between nanosheet particles and the nylon 610 matrix at high levels of nanofillers due to the agglomeration of the increased nanosheets within the polymer matrix. The aggregation of the nanosheets can also reduce the interlayer spacing and the empty space between the nanosheet particles in the polymer matrix [37,38]. This aggregation was corroborated by the SEM images shown in Section 3.4. This phenomenon is seen in all of the nanocomposites, whether incorporated with treated or untreated nanosheets.

### 3.3. FTIR Analysis

**Figure 10:**
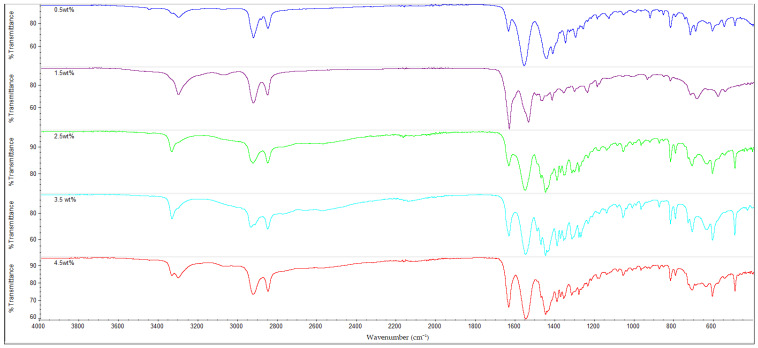
FTIR Spectra for Nylon 610 Nanocomposites with Graphite Nanosheets Treated at 200 °C and −0.1 MPa.

**Figure 11:**
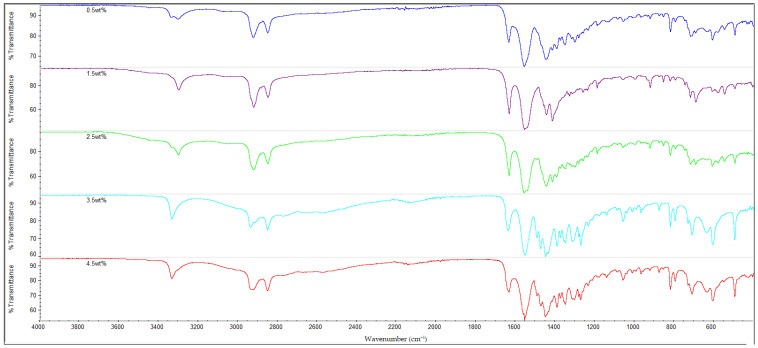
FTIR Spectra for Nylon 610 Nanocomposites with Graphite Nanosheets Treated at 175 °C and −0.1 MPa.

**Figure 12:**
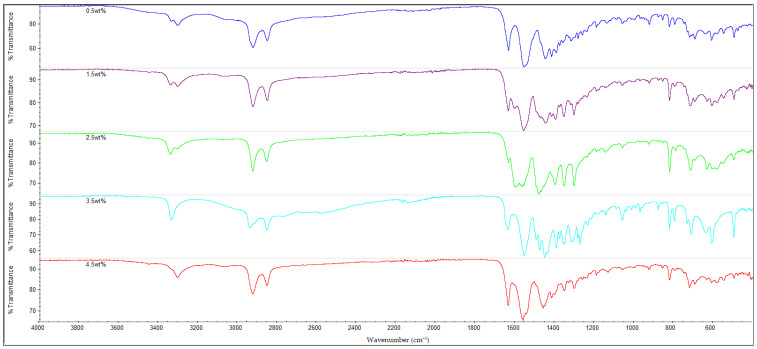
FTIR Spectra for Nylon 610 Nanocomposites with Graphite Nanosheets Treated at 150 °C and −0.1 MPa.

**Figure 13:**
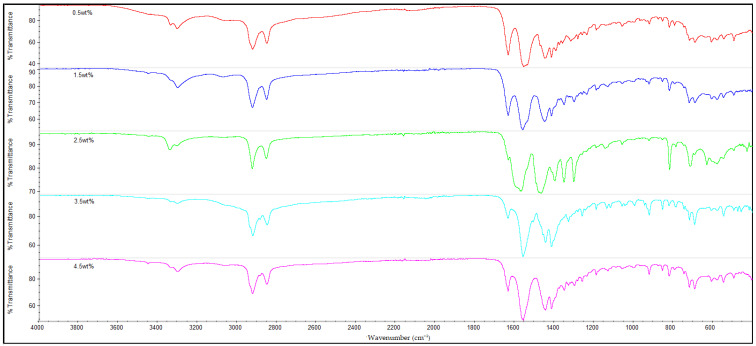
FTIR Spectra for Nylon 610 Nanocomposites with Untreated Graphite Nanosheets.

The infrared (IR) spectra of all the nylon 610 nanocomposites that were incorporated with treated or untreated graphite nanosheets of increasing wt% are displayed in Figure 10, Figure 11, Figure 12 and Figure 13. It can be seen that all of the 20 nanocomposites presented the characteristic IR bands of conventional nylon 610. For example, the appearance of an IR band around 3300 cm^−1^ is attributed to N-H stretching vibrations; the peaks detected around 2925 cm^−1^ and 2852 cm^−1^ signify the presence of C-H stretching bands in the methylene groups; the N-H bending and C-N stretching of amide II bands appear at approximately 1550 cm^−1^; the C=O stretching from the carbonyl groups and N-H stretching of an amide I band can be seen at around 1635 cm^−1^; and finally the presence of CH_2_ groups which represent the C-C backbone of the nylon polymer is shown at 1060 cm^−1^ and 1474 cm^−1^ [39,40]. Because the FTIR spectra of the nanocomposites are similar to that of nylon 610, it can be concluded that the addition of graphite nanosheets does not greatly affect the chemical structure of nylon 610. Furthermore, the FTIR spectra of nanocomposites incorporated with treated and untreated nanosheets do not differ much from one another, which also suggests that the presence of oxygen-containing functional groups on treated nanosheets might not affect the structure of the polymer matrix to a great extent.

As mentioned above, the IR band at 2925 cm^−1^ indicates the presence of C-H bonds. In the case of the nylon 610 nanocomposites that have incorporated treated nanosheets, the increase in loading levels of nanosheets treated at 200 °C and in a vacuum from 0.5 to 3.5 wt% led to a marginal increase of the wavenumber for C-H bond stretching before it decreased at 4.5 wt%, as seen in Table 4. This signifies that treated nanosheet particles and nylon 610 macromolecular chains had good interaction with one another inside the polymer matrix. The reason behind this is that the mostly non-polar treated nanosheets (excluding some oxygen-containing functional groups on their surfaces) are able to interact fittingly with the non-polar saturated C-H bonds of the nylon 610 polymer chains, which gives rise to the C-H stretching bonds found in the FTIR spectra [41]. However, when the loading level rises to 4.5 wt%, the wavenumber of the C-H bonds is found to drop from 2937.12 cm^−1^ to 2926.33 cm^−1^. This is because at the highest loading level, it is inevitable that some of the nanosheet particles will agglomerate to form larger particles. This results in the aggregated carbon nanofillers having a weaker interaction effect with the nylon 610 matrix, which leads to a reduction in the wavenumber of the C-H stretching, as the intermolecular vibrations within the polymer matrix have changed. This is due to the fact that the position of a peak in the IR band is related to the mass of the atoms within a certain bond and the strength of that bond, and the stronger the bond, the higher the frequency. This phenomenon also ties in with the decrease in conductivity at high loading levels of nanofillers due to agglomeration of the nanosheets. An identical phenomenon was also found in the nanosheets treated at 150 °C and −0.1 MPa. On the other hand, while the wavenumber for C-H bond stretching also decreased from 3.5 wt% to 4.5 wt% in the case of nanosheets treated at 175 °C, the wavenumber decreased from 0.5 wt% to 2.5 wt% instead of increasing. The reason behind this might be that the agglomeration of the nanosheets begins at a lower loading levels when compared with nanosheets treated at 200 °C and 150 °C.

The nanocomposites that used untreated nanosheets as filler also showed a similar pattern for C-H stretching. This is seen in Table 4, with the increasing wt% of the nanosheets leading to a gradual increase in the wavenumber for C-H stretching until 2.5 wt%, before the wavenumber decreases at 3.5 wt% (2924.11 cm^−1^) and at 4.5 wt% (2923.53 cm^−1^).

**Figure 14:**
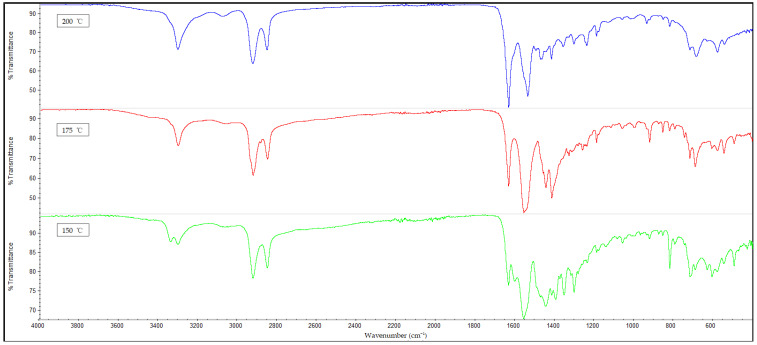
FTIR Spectra for Nylon 610 Nanocomposites with 1.5 wt% Graphite Nanosheets Treated at All Temperatures.

Additionally, FTIR spectroscopy revealed the presence of amide peaks at 1635 cm^−1^, 1550 cm^−1^ and 1190 cm^−1^ (amide I, II and III respectively) [42]. These amide peaks can be used to determine the extent of hydrogen bonding between the hydrogen atoms of nylon 610 and the oxygen-containing functional groups on the surfaces of treated nanosheets. This is accomplished by comparing the intensities of the amide peaks with one another and analysing the changes. For instance, it is shown that the nanocomposites with untreated nanosheets have amide peaks at 1635 cm^−1^ and 1550 cm^−1^ and have slightly lower intensity when compared with those incorporated with treated nanosheets. This is in agreement with the fact that supercritical water treatment is known to promote oxygen functionalities on the surfaces of carbon nanofillers, which facilitate the formation of hydrogen bonds between them and the hydrogen atoms in the nylon 610 matrix. Thus, these features demonstrate the hydrogen bonding between the nanofillers and the polymer, which is also beneficial to the conductivity of the nanocomposites, as stated in Section 3.1 [13]. Furthermore, Figure 14, which displays the FTIR spectra of nylon 610 nanocomposites with 1.5 wt% nanosheets treated at all temperatures of supercritical water treatment, reveals an increase in intensity of amide peaks (at 1635 cm^−1^) in relation with the temperature of the water treatment. This indicates an increase in hydrogen bonding between the oxygen-containing functional groups on the nanosheets and the hydrogen atoms of the polymer. Thus, it can be said that the rise in temperature of the water treatment resulted an increase in oxygen functionalities on the surfaces of the nanofillers. This is in line with the observations in Section 3.1.

### 3.4. SEM Analysis

**Figure 15:**
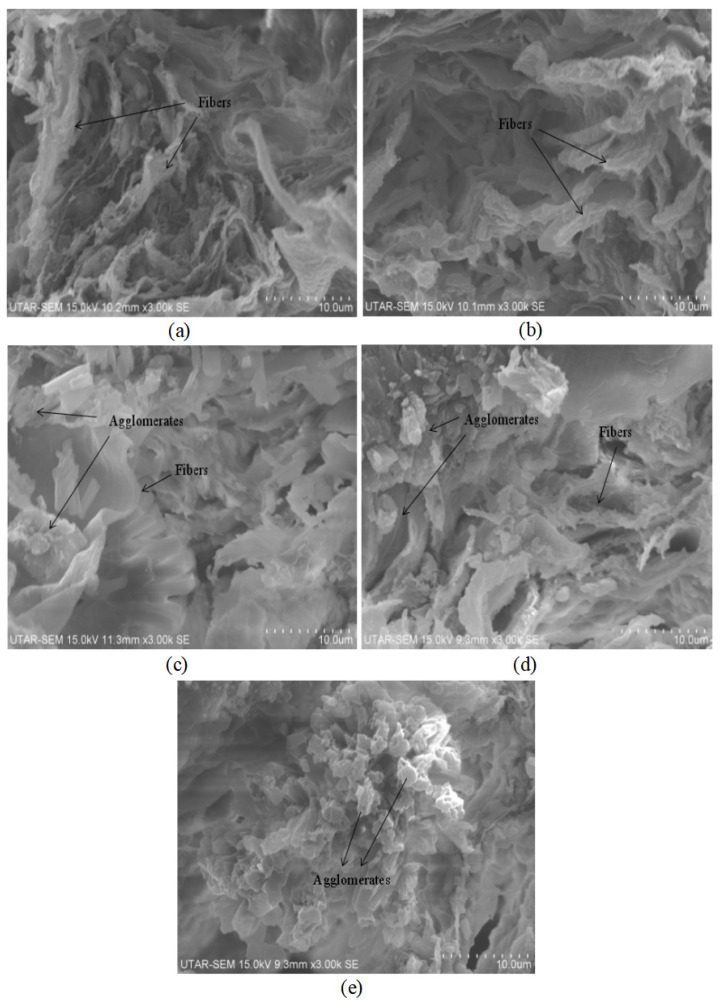
SEM Image of Graphite Nanosheets Treated at 200 °C and −0.1 MPa/Nylon 610 Nanocomposite at (**a**) 0.5 wt%, (**b**) 1.5 wt%, (**c**) 2.5 wt%, (**d**) 3.5 wt%, (**e**) 4.5wt% at ×3000 magnification.

The SEM images from Figure 15 depict the fractured surface morphologies of the treated graphite nanosheets (at 200 °C and −0.1 MPa)/nylon 610 nanocomposites from 0.5 wt% to 4.5 wt%. From the figures below, it is shown that the structures of all of the nanocomposites consist of a network of fibres with a rough surface that are interconnected with one another. Firstly, when the loading level of the treated nanosheets was low (0.5 wt% and 1.5 wt%), the SEM images revealed good matrix continuity, as the morphology of the composites only consisted of rough fibres, which also suggests a homogenous dispersion of the nanosheets, leading to better conductivity. However, as the amount of treated nanosheets increased, it was observed that agglomerates started to form on the surfaces of the fibres. This indicates that increasing the loading levels of the nanosheets can result in the agglomeration of the nanosheet particles in the nylon 610 matrix. This is especially the case for the highest loading level of treated nanosheets (4.5 wt%), as the surface of the nylon fibres is covered in clumps of large aggregates, as seen in Figure 15e. This agglomeration of the nanosheets is responsible for the decrease in conductivity for all the nanocomposites with treated nanosheets as nanofillers, as shown in Figure 3. This is because the paths for electron transport decrease when the conductive fillers are aggregated together. 

The fracture surface morphologies of the nanocomposites with treated and untreated nanosheets do not seem to differ much from one another, as both of them show a network of rough fibres and the surfacing of agglomerates with an increasing loading level of nanosheets. However, as shown in Figure 3, the conductivity of nanocomposites incorporated with untreated nanosheets is higher at higher wt% when compared with those combined with treated nanosheets. The reason behind this is the formation of oxygen-containing functional groups on the surfaces of treated nanosheets due to the supercritical water treatment, as the high amount of treated nanosheets in the nylon 610 matrix may result in them forming bonds with one another rather than with the polymer matrix, leading to agglomeration. By contrast, the lack of oxygen functionalities on the surfaces of untreated nanosheets decreases the probability of them aggregating with one another and allows some of them to be distributed within the nylon 610 matrix, which results in a higher conductivity at higher wt%.

It is also important to note that the SEM images below show a lack of nanosheets, owing to the small amounts of nanosheets comparatively and their small size (2–10 nm). This information is related to the conductivity of the nanocomposites, as the geometry or size of the conductive fillers will influence the percolation threshold of a material. For instance, nanofillers with an elongated shape like fibres or sheets possess higher aspect ratios (ratio of length to width) compared to spherical fillers, which provides a larger advantage in forming conductive networks because a larger aspect ratio leads to more interparticle contact [43]. This explains why the addition of graphite nanosheets (treated or untreated), which have a small size and a high aspect ratio, results in a large increase in conductivity when compared to pure nylon.

## 4. Conclusions

The results show that semi-conductive graphite nanosheets/nylon 610 composites have been formed using an environmentally friendly method of treatment for the nanofillers. The synthesis of the nanocomposites using interfacial polymerization is also a fast and convenient method. This is shown by all of the nanocomposites having conductivities many times that of neat nylon 610 (10^−14^ S/m). XRD analysis has also shown that the crystal structure of the nanofillers before and after supercritical water treatment remain unchanged. This is beneficial to the overall conductivity of the nanocomposite, as graphite nanosheets are inherently conductive. Furthermore, FTIR spectra also revealed the presence of amide peaks, which is indicative of hydrogen bonding between the nanosheets and the polymer matrix. It is also shown that the intensities of the amide peaks for the nanocomposites with treated nanosheets are slightly higher than those of the nanocomposites with untreated nanosheets. This is due to the increase in oxygen-containing functional groups that were formed after the water treatment.

Additionally, nanocomposites with treated nanosheets have better conductivity at lower wt%, while composites with untreated nanosheets exhibit the opposite behaviour. This is because an increase in filler concentration leads to a higher chance of forming a conductive network, as the conductive fillers have a higher chance to be in contact with each other. This is shown by the incorporation of 4.5 wt% untreated nanosheets resulting in the second-highest conductivity (0.004110 S/m). However, the opposite can be seen occurring for the incorporation of treated nanosheets. The reason for this might be the change in the surface morphology of the treated nanofillers due to the formation of oxygen-containing functional groups on their surfaces, which affects the conductivity of the composite. Another reason for this trend is the aggregation of the nanofillers due to the high content of fillers in the polymer matrix. Such agglomeration can prevent the nanofillers from contacting one another, hindering electrons from flowing freely within the nanocomposite. This is shown by the lowest wt% of treated nanosheets recording the highest conductivity value (0.004135 S/m). In conclusion, this improvement in the electric properties can be attributed to the intact crystal structure of the nanosheets and the interaction between the nanofillers and the nylon 610 matrix.

It can also be concluded that the supercritical water treatment used to functionalize the surfaces of the graphite nanosheets is only efficient if the temperature is sufficiently high. Once the temperature of the water is adequate, only a small amount of nanofillers is needed to attain a high conductivity. Thus, the nylon 610 nanocomposite incorporated with 0.5 wt% graphite nanosheets treated at 200 °C and −0.1 MPa shows the highest potential for the development of new electrically conductive nanomaterials. On the other hand, while the nanocomposite with 4.5 wt% untreated nanosheets has the second-highest conductivity, it is deemed not very economical, as it requires relatively high amounts of nanosheets.

## Figures and Tables

**Table 1:** Average Conductivity of All the Nylon 610/Graphite Nanosheets Nanocomposites.

Type of Nanofiller Incorporated in Nanocomposite	Loading Levels of Nanofiller (wt%)	Average Conductivity (S/m)
Graphite Nanosheets Treated at 200 °C and −0.1 MPa	0.5	0.004135
	1.5	0.003441
	2.5	0.003647
	3.5	0.003678
	4.5	0.003559
Graphite Nanosheets Treated at 175 °C and −0.1 MPa	0.5	0.002007
	1.5	0.002212
	2.5	0.002370
	3.5	0.001880
	4.5	0.001918
Graphite Nanosheets Treated at 150 °C and −0.1 MPa	0.5	0.002536
	1.5	0.002305
	2.5	0.003282
	3.5	0.002788
	4.5	0.001974
Untreated Graphite Nanosheets	0.5	0.003490
	1.5	0.003115
	2.5	0.003640
	3.5	0.003817
	4.5	0.004110

**Table 2:** *d*-Spacing and Crystallite Size for All 2*θ* = 24.6° Peaks in Treated and Untreated Nanosheets.

Sample	*d*-Spacing, Å	Inter-Chain Separation (*R*), Å
Treated Graphite Nanosheets	3.6597	4.5747
Untreated Graphite Nanosheets	3.6105	4.5132

**Table 3:** *d*-Spacing and Crystallite Size for All 2*θ* = 24.3° Peaks in Graphite Nanosheets/Nylon 610 Nanocomposites.

Type of Nanofiller Incorporated in Nanocomposite	Loading Levels of Nanofiller (wt%)	*d*-Spacing, Å	Inter-Chain Separation (*R*), Å
Graphite Nanosheets Treated at 200 °C and −0.1 MPa	0.5	3.63859	4.55238
	1.5	3.65959	4.57865
	2.5	3.64372	4.55879
	3.5	3.62868	4.53998
	4.5	3.65608	4.57425
Graphite Nanosheets Treated at 175 °C and −0.1 MPa	0.5	3.67776	4.60138
	1.5	3.64804	4.56420
	2.5	3.69221	4.61945
	3.5	3.64804	4.56120
	4.5	3.70190	4.63158
Graphite Nanosheets Treated at 150 °C and −0.1 MPa	0.5	3.65589	4.57401
	1.5	3.77629	4.62399
	2.5	3.66284	4.58271
	3.5	3.67567	4.59876
	4.5	3.70494	4.63538
Untreated Graphite Nanosheets	0.5	3.66284	4.58271
	1.5	3.69886	4.62778
	2.5	3.70190	4.63158
	3.5	3.64804	4.56420
	4.5	3.61592	4.52400

**Table 4:** Wavenumbers of C-H Stretching Type for All of the Graphite Nanosheets/Nylon 610 Nanocomposites.

Type of Nanofiller Incorporated in Nanocomposite	Loading Level of Graphite Nanosheets, wt%	Wavenumber, cm^−1^
		C-H Stretching
Graphite Nanosheets Treated at 200 °C and −0.1 MPa	0.5	2924.37
	1.5	2924.50
	2.5	2926.37
	3.5	2937.12
	4.5	2926.33
Graphite Nanosheets Treated at 175 °C and −0.1 MPa	0.5	2925.15
	1.5	2922.99
	2.5	2922.98
	3.5	2938.34
	4.5	2926.16
Graphite Nanosheets Treated at 150 °C and −0.1 MPa	0.5	2923.61
	1.5	2924.08
	2.5	2924.55
	3.5	2938.36
	4.5	2924.18
Untreated Graphite Nanosheets	0.5	2924.18
	1.5	2924.04
	2.5	2924.76
	3.5	2924.11
	4.5	2923.53

## Data Availability

Not applicable.

## Abbreviations

XRD, X-ray diffraction; FTIR, Fourier transform infrared spectroscopy; SEM, scanning electron microscopy; CO_2_, carbon dioxide; HMDA, hexamethylenediamine; NaOH, sodium hydroxide; ATR, attenuated total reflectance; IR, infrared.

## Appendix A

This appendix shows the bar chart of the conductivities for all 20 of the graphite nanosheets/nylon 610 nanocomposites. The purpose of this is to show the error bars for the conductivities measured. All of the results are the same as in Figure 3.

The tables below show the resistance and conductivity data for each of the five samples for all nanocomposites synthesized. The conductivity is then obtained by calculating the average from the conductivity data and rounding it up to 4 significant figures, which is found in Table 1.

**Table A1:** Conductivity Data for Nylon 610 Nanocomposites with 0.5 wt% Graphite Nanosheets Treated at 200 °C and −1 bar.

Sample	1	2	3	4	5
Resistance (ohm)	22,902.51471	22,902.51471	27,066.60829	29,773.26912	29,773.26912
Conductivity (S/m)	0.005352047	0.00507036	0.00407579	0.003528822	0.002646617

**Table A2:** Conductivity Data for Nylon 610 Nanocomposites with 1.5 wt% Graphite Nanosheets Treated at 200 °C and −1 bar.

Sample	1	2	3	4	5
Resistance (ohm)	33,081.41014	33,081.41014	33,081.41014	29,773.26912	29,773.26912
Conductivity (S/m)	0.002778947	0.002381955	0.003510249	0.003900277	0.004631579

**Table A3:** Conductivity Data for Nylon 610 Nanocomposites with 2.5 wt% Graphite Nanosheets Treated at 200 °C and −1 bar.

Sample	1	2	3	4	5
Resistance (ohm)	33,081.41014	33,081.41014	33,081.41014	33,081.41014	29,772.36911
Conductivity (S/m)	0.003334737	0.003510249	0.002899771	0.003705263	0.0049405

**Table A4:** Conductivity Data for Nylon 610 Nanocomposites with 3.5 wt% Graphite Nanosheets Treated at 200 °C and −1 bar.

Sample	1	2	3	4	5
Resistance (ohm)	33,081.41014	29,773.26912	29,772.36911	33,081.41014	33,081.41014
Conductivity (S/m)	0.002778947	0.002744639	0.003900395	0.00392322	0.004446316

**Table A5:** Conductivity Data for Nylon 610 Nanocomposites with 4.5 wt% Graphite Nanosheets Treated at 200 °C and −1 bar.

Sample	1	2	3	4	5
Resistance (ohm)	33,081.41014	33,081.41014	33,081.41014	33,081.41014	29,772.36911
Conductivity (S/m)	0.003334737	0.003510249	0.002899771	0.003705263	0.0049405

**Table A6:** Conductivity Data for Nylon 610 Nanocomposites with 0.5 wt% Graphite Nanosheets Treated at 175 °C and −1 bar.

Sample	1	2	3	4	5
Resistance (ohm)	37,216.5864	42,533.2416	42,533.2416	42,533.2416	49,622.1152
Conductivity (S/m)	0.002694737	0.002255378	0.002074947	0.001621053	0.001389474

**Table A7:** Conductivity Data for Nylon 610 Nanocomposites with 1.5 wt% Graphite Nanosheets Treated at 175 °C and −1 bar.

Sample	1	2	3	4	5
Resistance (ohm)	42,533.2416	49,622.1152	42,533.2416	49,622.1152	42,533.2416
Conductivity (S/m)	0.003242105	0.001933181	0.002074947	0.001646784	0.002161404

**Table A8:** Conductivity Data for Nylon 610 Nanocomposites with 2.5 wt% Graphite Nanosheets Treated at 175 °C and −1 bar.

Sample	1	2	3	4	5
Resistance (ohm)	42,533.2416	49,622.1152	49,622.1152	42,533.2416	49,622.1152
Conductivity (S/m)	0.002255378	0.001482105	0.002223158	0.002470176	0.003420243

**Table A9:** Conductivity Data for Nylon 610 Nanocomposites with 3.5 wt% Graphite Nanosheets Treated at 175 °C and −1 bar.

Sample	1	2	3	4	5
Resistance (ohm)	49,622.1152	49,622.1152	49,622.1152	49,622.1152	49,622.1152
Conductivity (S/m)	0.002470176	0.001482105	0.002021053	0.002117293	0.00130774

**Table A10:** Conductivity Data for Nylon 610 Nanocomposites with 4.5 wt% Graphite Nanosheets Treated at 175 °C and −1 bar.

Sample	1	2	3	4	5
Resistance (ohm)	42,533.2416	49,622.1152	49,622.1152	42,533.2416	49,622.1152
Conductivity (S/m)	0.002881871	0.001111579	0.001778526	0.002255378	0.00158797

**Table A11:** Conductivity Data for Nylon 610 Nanocomposites with 0.5 wt% Graphite Nanosheets Treated at 150 °C and −1 bar.

Sample	1	2	3	4	5
Resistance (ohm)	37,216.5864	37,216.5864	37,216.5864	33,081.41014	3,3081.41014
Conductivity (S/m)	0.00197614	0.002117293	0.002044283	0.003031579	0.003510249

**Table A12:** Conductivity Data for Nylon 610 Nanocomposites with 1.5 wt% Graphite Nanosheets Treated at 150 °C and −1 bar.

Sample	1	2	3	4	5
Resistance (ohm)	29,773.26912	37,216.5864	33,081.41014	37,216.5864	33,081.41014
Conductivity (S/m)	0.002744639	0.002117293	0.002381955	0.002195712	0.002084211

**Table A13:** Conductivity Data for Nylon 610 Nanocomposites with 2.5 wt% Graphite Nanosheets Treated at 150 °C and −1 bar.

Sample	1	2	3	4	5
Resistance (ohm)	17,513.68772	24,811.0576	29,773.26912	37,216.5864	37,216.5864
Conductivity (S/m)	0.004845344	0.003705263	0.002850203	0.002964211	0.002044283

**Table A14:** Conductivity Data for Nylon 610 Nanocomposites with 3.5 wt% Graphite Nanosheets Treated at 150 °C and −1 bar.

Sample	1	2	3	4	5
Resistance (ohm)	42,533.2416	42,533.2416	49,622.1152	42,533.2416	42,533.2416
Conductivity (S/m)	0.001995142	0.002255378	0.001710122	0.003990284	0.003990284

**Table A15:** Conductivity Data for Nylon 610 Nanocomposites with 4.5 wt% Graphite Nanosheets Treated at 150 °C and −1 bar.

Sample	1	2	3	4	5
Resistance (ohm)	42,533.2416	42,533.2416	42,533.2416	42,533.2416	42,533.2416
Conductivity (S/m)	0.002074947	0.001621053	0.001852632	0.002593684	0.001729123

**Table A16:** Conductivity Data for Nylon 610 Nanocomposites with 0.5 wt% Untreated Graphite Nanosheets.

Sample	1	2	3	4	5
Resistance (ohm)	27,066.60829	27,065.7901	29,773.26912	29,773.26912	29,773.26912
Conductivity (S/m)	0.003705263	0.003881822	0.003528822	0.003368421	0.002964211

**Table A17:** Conductivity Data for Nylon 610 Nanocomposites with 1.5 wt% Untreated Graphite Nanosheets.

Sample	1	2	3	4	5
Resistance (ohm)	29,773.26912	33,081.41014	29,773.26912	33,081.41014	33,081.41014
Conductivity (S/m)	0.003900277	0.002778947	0.002964211	0.003031579	0.002899771

**Table A18:** Conductivity Data for Nylon 610 Nanocomposites with 2.5 wt% Untreated Graphite Nanosheets.

Sample	1	2	3	4	5
Resistance (ohm)	33,081.41014	33,081.41014	33,081.41014	33,081.41014	33,081.41014
Conductivity (S/m)	0.004168421	0.002899771	0.003510249	0.00317594	0.004446316

**Table A19:** Conductivity Data for Nylon 610 Nanocomposites with 3.5 wt% Untreated Graphite Nanosheets.

Sample	1	2	3	4	5
Resistance (ohm)	33,081.41014	33,081.41014	29,773.26912	33,081.41014	29,773.26912
Conductivity (S/m)	0.004168421	0.002899771	0.003900277	0.00317594	0.004940351

**Table A20:** Conductivity Data for Nylon 610 Nanocomposites with 4.5 wt% Untreated Graphite Nanosheets.

Sample	1	2	3	4	5
Resistance (ohm)	29,773.26912	33,081.41014	29,773.26912	29,773.26912	29,773.26912
Conductivity (S/m)	0.004940351	0.004168421	0.002964211	0.004359133	0.004116959
